# Mediastinal Pancreatic Pseudocyst with Hemoptysis – A Thoracic Complication of Pancreatitis

**DOI:** 10.7759/cureus.11518

**Published:** 2020-11-17

**Authors:** Arpád Panyko, Marián Vician, Martin Dubovský, Rudolf Škubla

**Affiliations:** 1 Surgery, University Hospital Bratislava, Bratislava, SVK; 2 Medicine, Comenius University of Bratislava, Bratislava, SVK

**Keywords:** hemoptysis, pancreatic fistula, mediastinal cyst, pancreatic pseudocyst, pancreatitis, extra-pancreatic pseudocyst

## Abstract

Mediastinal pancreatic pseudocysts are rarely encountered complications of pancreatic diseases. Pseudocysts most often expand into surrounding structures, just rarely into the mediastinum. Usually, they present with abdominal pain, and the symptoms correlate with the location of the pseudocysts. We describe a case of a pancreatic pseudocyst that penetrated the thoracic cavity through the diaphragm and set up a communication with the bronchial tree developing an episode of massive hemoptysis. This case is of particular interest because just a few similar cases were published before. Based on this report, we emphasize the need for early accurate diagnosis; surgeons should maintain a higher index of suspicion for mediastinal pancreatic pseudocyst in patients with chronic pancreatitis.

## Introduction

Pancreatic pseudocysts are circumscribed collections of fluid rich in pancreatic enzymes, blood and necrotic tissue. They are relatively common complications of chronic pancreatitis. Most often, they expand into surrounding structures, but sometimes can be localized in unusual areas, one such is the mediastinum. Mediastinal pancreatic pseudocyst (MPP) is a rare finding, often presenting with atypical symptoms [1]. This report is of particular interest because just a few cases of MPP presenting with hemoptysis have been published in the world literature [2]. Clinically meaningful are all the symptoms and the response, which occurs during the progression into the thoracic cavity. This unique complication suggests that maybe a common medical problem (e.g., hiatal hernia) allows the expansion of the pancreatic pseudocyst.

## Case presentation

We present the case of a 59-year-old patient with a known history of chronic obstructive pulmonary disease and chronic pancreatitis due to alcohol abuse. In the past, he has been repeatedly examined for various respiratory and intermittent dyspeptic disorders. A pneumologist initially treated him at the outpatient department. A chest X-ray showed bilateral pleural effusion (massive on the right side) for which antibiotic treatment was started. Due to dyspnea, new episodes of massive hemoptysis and ineffective outpatient treatment, he was admitted to our hospital.

**Figure 1:**
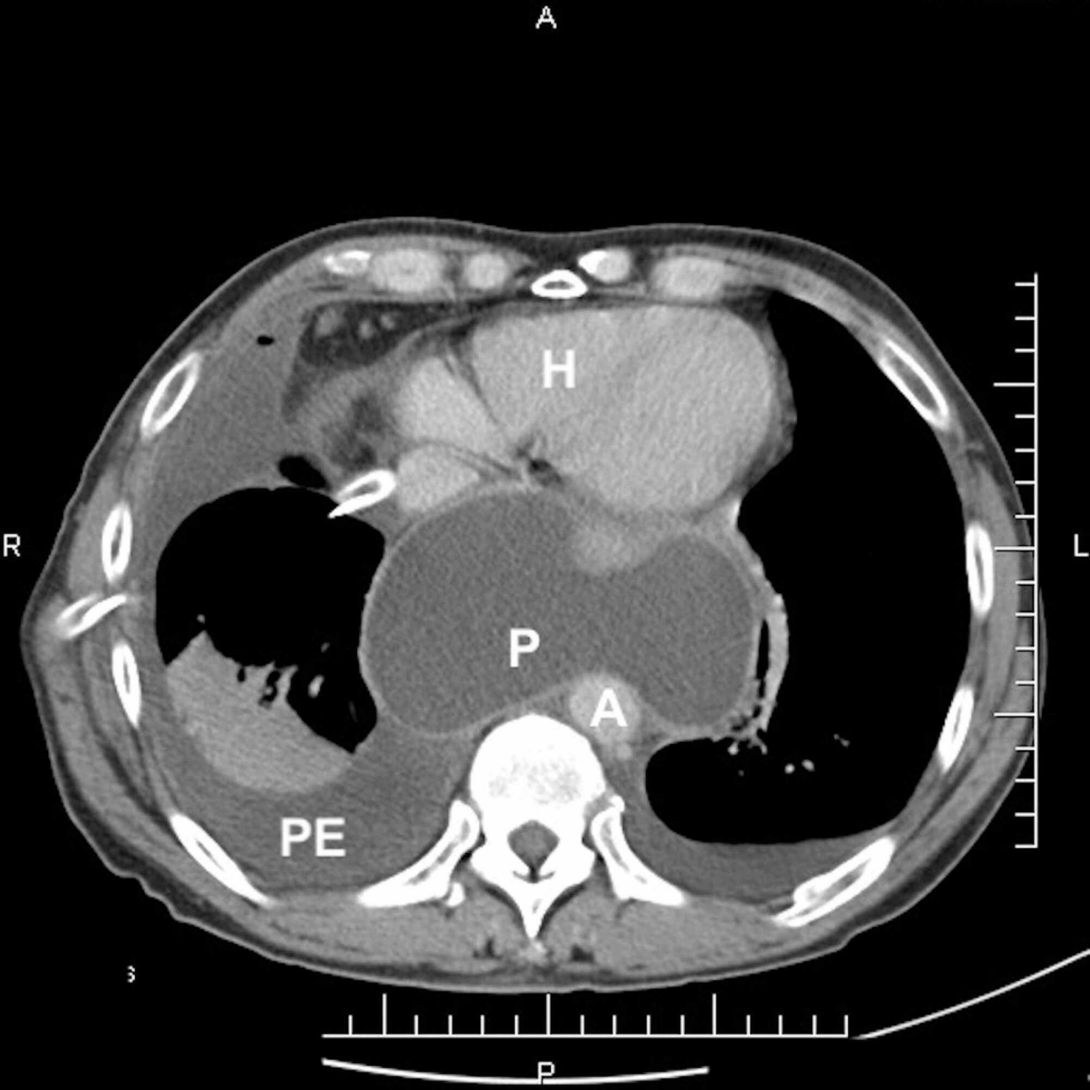
Chest CT scan (axial plane) at the level of the lower mediastinum reveals a mass (P- pseudocyst) and right sided pleural effusion (PE). Heart (H), aorta (A).

**Figure 2:**
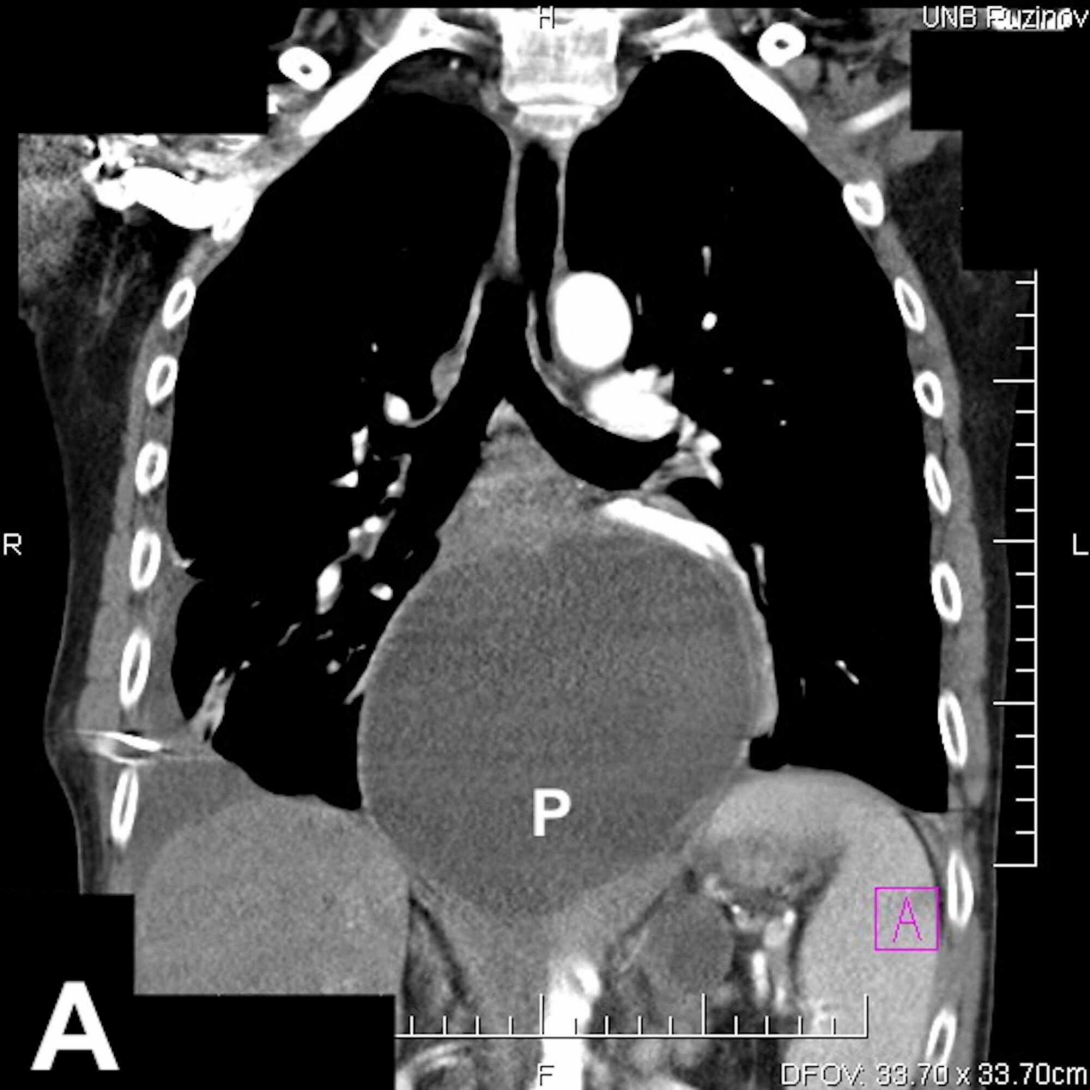
Chest CT scan (coronal) reveals a pseudocyst (P) extending from the body and the tail of the pancreas to the posterior mediastinum.

**Figure 3:**
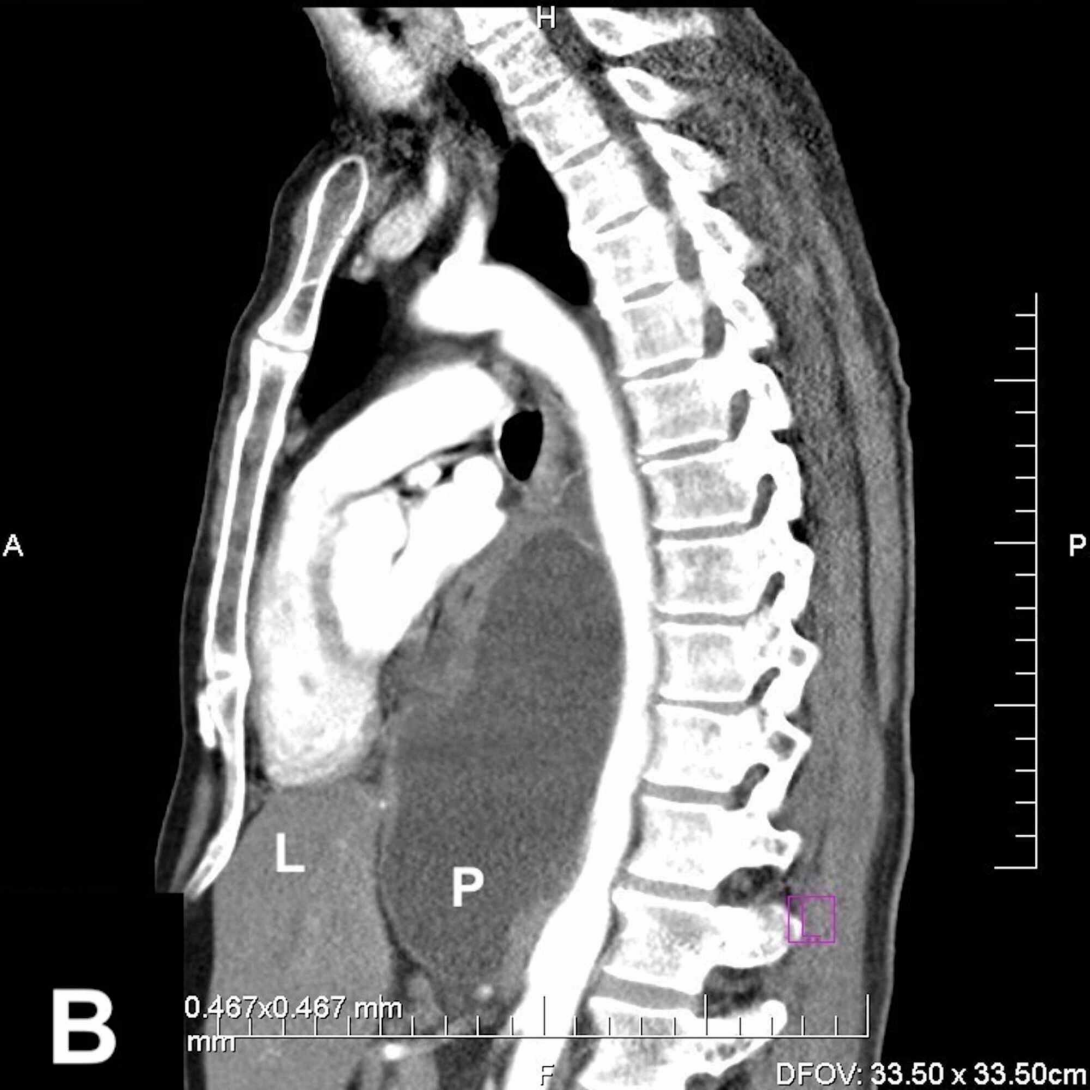
Chest CT scan (sagittal) demonstrate a longitudinal fluid chollection (P-pseudocyst) in the mediastinum, along the thoracic aorta. (L-liver)

On admission, his laboratory results showed normal white blood cell count (9,2 x10^9^/l) and elevated C-reactive protein (40 mg/l). Pancreatic enzymes were also elevated - amylase 4.21 ukat/l (normal range 0-1.67 ukat/L), lipase 2.28 ukat/l ( normal range 0-1.0 ukat/l). Bronchoscopy did not show any remarkable findings or exact cause of hemoptysis. Due to massive right-sided fluidothorax, a pleural fluid aspiration was carried out. We initially removed 400ml brownish fluid and the samples were sent for analysis. Chest X-ray revealed only slight regression of the effusion in the right hemithorax; simultaneously, a cystic lesion was also unmasked. The previous chest fluid biochemistry analysis showed high amylase levels (12.3 ukat/l), suspecting the lesion to be a pancreatic pseudocyst. The pleural fluid showed no evidence of infection or malignancy. Chest and abdominal computed tomography (CT) examination excluded primary pulmonary disease, but revealed a surprising finding of an extensive cystic formation in the lesser sac with connection to the pancreas and extending to the posterior mediastinum, with compression of the heart and deviation of the esophagus (Figures 1-3). The fluid collection had an enhanced wall and was associated with chronic pancreatitis. The bulk of pseudocyst was in the mediastinum and was probably the cause of the right-sided pleural effusion. It also set up an intimate communication with the bronchial tree leading to hemoptysis episodes. Oncomarkers carbohydrate antigen 19-9 (CA 19-9), squamous cell carcinoma antigen (SCC), neuron-specific enolase (NSE), cytokeratin 19 fragment (CYFRA) were within normal values, but carcinoembryonic antigen in the serum was elevated (4.32 ug/l). 

**Figure 4:**
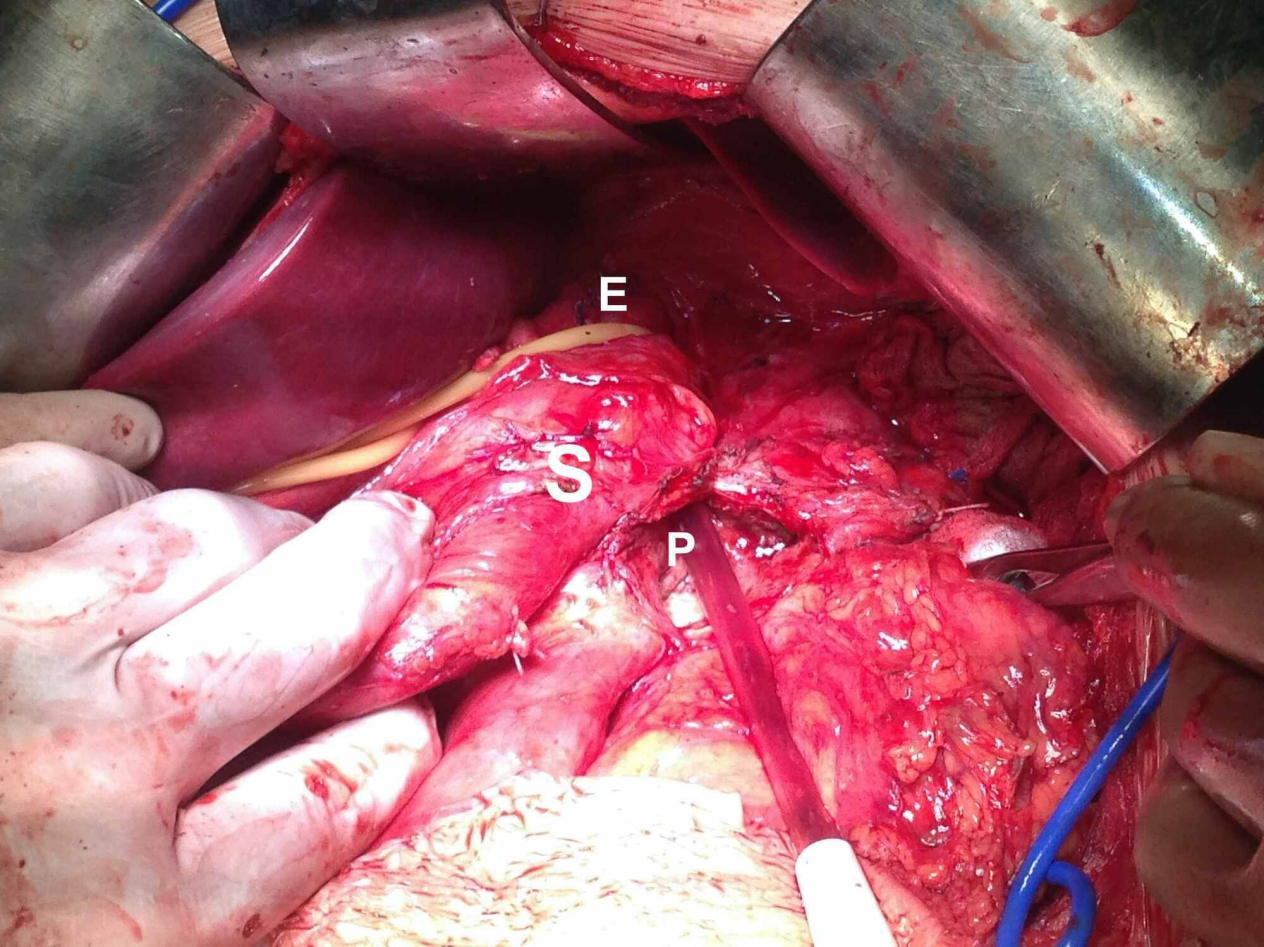
Intraoperative pictures: clearly visible opening of the pseudocyst. stomach – S, pseudocyst – P, esophagus – E, diaphragm – D

**Figure 5:**
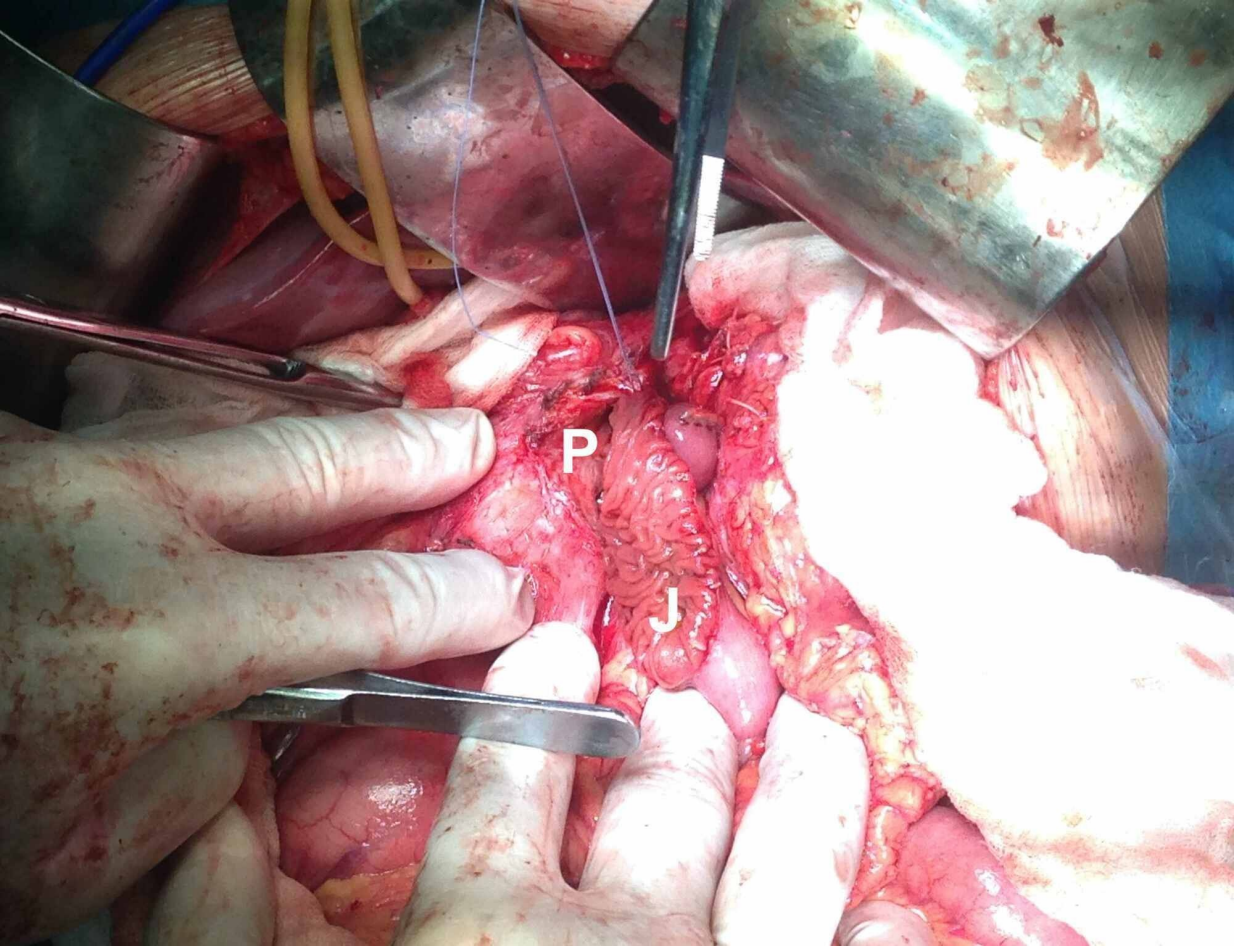
Intraoperative pictures: open surgical drainage - constructing the pseudocysto-jejuno-anastomosis. pseudocyst – P, jejunum - J

After this short hospitalization at the pulmonary department, he was admitted to our department. Further evaluation with magnetic resonance cholangiopancreatography (MRCP) showed a significant progression in the pseudocyst's size with dorsal propagation. In the posterior lower mediastinum, we observed an encapsulated "8" -shaped fluid collection measuring 110x65mm, compressing the esophagus. Pseudocyst contained two more solid wall zones of residual necrotic pancreatic tissue or blood clots. The intra- and extrahepatic bile ducts were without dilatation. Given the above finding and the failure of conservative treatment and endoscopic drainage by ERCP (endoscopic retrograde cholangiopancreatography), he was scheduled for elective surgery. On the operation, extensive adhesions were found between the lesser sac, the stomach, and the spleen after previous attacks of pancreatitis. A sizeable pancreatic pseudocyst was found after careful adhesiolysis, as described on the CT and MR scans. We verified the passage to the chest through the esophageal hiatus (Figure 4). The construction of a gastrocystostomy was not possible as it would cause stenosis of the gastro-esophageal junction. We decided to construct a pseudocyst-jejunal-anastomosis en Roux-Y. Roux-en-Y loop of jejunum was brought retrocolic and retrogastric up to the pseudocyst. The wall of the pseudocyst was sufficiently mature for a side-to-side anastomosis (Figure 5). The patient's postoperative recovery was uneventful. The patient was released to outpatient care on the 10th postoperative day. After discharge from the hospital, the patient continued taking digestive enzymes. After three months, the follow-up CT scan revealed a significant resolution of the pseudocyst.

## Discussion

Mediastinal pancreatic pseudocyst has been reported in less than 1% of all pseudocysts. Alcohol abuse is the second most common cause of AP after gallstones [3]. Recurrent attacks of acute pancreatitis lead to the disruption of the pancreatic duct. The pancreatic fluid is usually confined in the retroperitoneal space, but can occasionally slip into the posterior mediastinum from the oesophageal or the aortic hiatus, leading to the formation of a pseudocyst. Symptoms may vary from dysphagia, dyspnea, cough, hemoptysis, pleural effusions, chest pain [3]. The challenge in the management of mediastinal pancreatic pseudocyst lies in its rarity and complex pathophysiology. Diet, enzyme supplementation, abstinence of alcohol should be the main goals of conservative treatment in all patients [4]. Further treatment depends on the size, number, location, relationship to the adjacent anatomical structures, severity of symptoms, the presence of infection, and communication of the pseudocyst with the pancreatic duct [5]. As demonstrated in our case report, the unexpected locations of the extensive pseudocyst, chronic pancreatitis, and pulmonary complications all contribute to morbidity. Spontaneous regression has been described in the literature but is very rare in a pseudocyst of this size. Imaging methods (CT, MR) have an essential place and are most commonly used in determining an accurate diagnosis and the exact location. MRCP can also confirm the disruption of the pancreatic duct. The principle of treatment is drainage of pancreatic fluid using various techniques. Recent advances in endoscopic and radio intervention techniques have shown promising results; not only are a useful adjunct to surgical treatment, but in some cases, they can completely replace it [6, 7, 8, 9, 10]. However, their limited availability limits their widespread use, and management of complex mediastinal pseudocyst may still require open surgical drainage procedures. Further studies are needed on the safety and efficacy of these mini-invasive techniques. Relapses are common, and complications can be severe [11]. Open surgical treatment is now less frequent and should be reserved for the most resistant cases in patients unable to undergo laparoscopic surgery [11]. At the moment, there are no guidelines for treatment; intervention is individually tailored [12]. Multidisciplinary care is essential to provide the best care possible.

## Conclusions

Mediastinal extension of pancreatic pseudocysts are present with atypical symptoms due to their unique location. When it comes to proper diagnosis and management, they are still a challenge for gastroenterologists and surgeons. This report emphasizes the need for early accurate diagnosis and surgical treatment in symptomatic MPP. In patients with chronic pancreatitis and chronic pulmonary diseases, surgeons should retain a higher suspicion index for the mediastinal pancreatic pseudocyst.
